# Critical aspects of educating clinical management and clinical reasoning in primary teeth pulpotomy: A qualitative study based on the perspectives of experts and novices

**DOI:** 10.1111/eje.12710

**Published:** 2021-08-20

**Authors:** Fatemeh Janesarvatan, Hamidreza Hassanabadi, Saeedeh Mokhtari, Peter Van Rosmalen

**Affiliations:** ^1^ Department of Educational Development and Research SHE Maastricht University Maastricht The Netherlands; ^2^ Department of Educational Psychology Faculty of Psychology and Educational Science Kharazmi University Tehran Iran; ^3^ Department of Pediatric Dentistry School of Dentistry Tehran University of Medical Sciences Tehran Iran

**Keywords:** clinical management, clinical reasoning, dental education, dental students, simulation

## Abstract

**Introduction:**

In dental education, students must learn to integrate and coordinate complex knowledge, skills and attitudes and to transfer this learning to clinical practice. One major issue of concern in education in general and dental education, in particular, is to fill the gap between knowledge and practice.

**Methods:**

The purpose of this study was to explore the problems that dental students have in transferring knowledge from the classroom to the real clinical setting. More specifically, we investigated the factors that complicate clinical management and clinical reasoning for these novices, including their common errors, in order to design an educational simulation programme in primary teeth pulpotomy. To this end, we conducted 16 semi‐structured interviews with experts and novices, performing a thematic analysis of the data obtained. All interviews were audio recorded and transcribed verbatim.

**Results:**

For each major skill—clinical management and clinical reasoning—we identified complicating factors and common errors that related to the child (the patient), parents and dental student (the three main themes). For each theme, we identified further sub‐themes.

**Conclusion:**

The data obtained provided valuable insights into the factors that affect dental students' performance on clinical management and clinical reasoning in primary teeth pulpotomy.

## 1 INTRODUCTION

Dental education increasingly emphasises the integration of multiple skills, such as clinical management, decision‐making and communication skills in order to perform successfully and to provide oral health care for the benefit of the individual patient.^1^, ^2^, ^3^ Nevertheless, the process of transferring learning from theory to practice is a challenge in dental education. Students still experience many problems at their first encounter with real patients.^3^, ^4^, ^5^ They lack clinical experience due to an overemphasis on theory in undergraduate training. Ray et al.,^4^ for instance, observed that 80 per cent of final‐year dental students felt not ready for clinical practice in terms of diagnosing and treatment planning and lacked confidence in communication due to insufficient practice. Similarly, Ali et al.,^3^ found that students scored low on the ability to assess treatment needs and on treatment planning. Dental education aims to achieve this learning transfer either in the classroom or in clinical practice. However, teaching and learning in the practice setting will be typically implicit, limited and variable. Therefore, students may find that their education has not trained them sufficiently and that they need extra support.^5^, ^6^

New approaches to teaching and learning emphasise the use of authentic learning tasks; these tasks are a tool to help learners to promote the integration of knowledge, skills and attitudes, the coordination of constituent skills and the transfer of learning to real‐life situations.^7^, ^8^, ^9^ Walker et al.^10^ concluded that dental programmes must offer their students more complex dental scenarios involving a variety of communication skills in a secure and simulated environment. Case studies on the use of simulated environments in dental education^4^, ^11^, ^12^, ^13^ confirm the potential value.

Despite the fact that several studies have reported on the practical challenges^4^, ^6^ in dental education,^4^, ^14^, ^15^ qualitative studies on the perceptions of undergraduate preparation for independent general dental practice are scant. More specifically, no study has hitherto examined the factors that complicate clinical management and clinical reasoning, including the associated errors that are common amongst novices.

Our ultimate aim was to design an educational simulation package for novices in dentistry to offer them additional practice with the integration of knowledge, skills and attitudes. The purpose of this study, therefore, was to investigate the phenomenon associated with dental students and their experiences of learning towards becoming a dentist in order to answer the following research question:

- What aspects of practical importance are critical in dental students' learning of clinical management and clinical reasoning skills (in terms of complicating factors and common errors)?

## 2 METHODS

### 2.1 Design of the interview

This qualitative study is based on the Phenomenological approach, to illuminate a phenomenon through how it is perceived by the participants in a situation.^16^ We aimed to identify the factors that complicate clinical management and clinical reasoning including the errors that are common amongst dental students. As a first step, following the advice of two paediatric dentists, we decided to focus on pulp treatment as our object of study. The dentists were unanimous that the incidence of errors in this field was relatively high and that it was more difficult for most students than other treatments. Next, with their help, we designed a semi‐structured interview. Moreover, we reviewed the research literature and scientific documents from the American Pediatric Dental Association,^17^ the educational curriculum in paediatric dentistry, prerequisite and passed courses and required prior knowledge. Based on this scrutiny, we formulated the steps in clinical management and clinical reasoning that students should follow when writing the treatment plan.

In the second round, the two paediatric dentists reviewed the relevant steps and sub‐steps and confirmed the structure of the interview. The interview consisted of three sections: (1) an introduction to the research objectives and opening questions asking for demographic information and professional experience; (2) the main section with questions about the transfer of clinical management and clinical reasoning skills in primary teeth pulpotomy, respectively; and (3) concluding questions. More specifically, the interview aimed to find an answer to the following questions:

‐ How do students apply their clinical management and diagnostic skills in their interactions with the child (ie the patient) and the parent?
‐ What factors or characteristics (individual and dental) can complicate the diagnostic process?
‐ What errors can occur in the process?
‐ How do students receive feedback from their supervisors?
‐ Do students and experts believe it is feasible to train the process in a simulation programme?

### 2.2 Participants

Given the intention of this research, that is to gather data regarding the phenomenon of learning and working towards becoming a dentist, we decided to investigate not only the students' but also the experts' perspective. This is to ensure the validity^16^ of our findings since the students we focus on have limited practical experience. For both groups, we used purposive sampling.^18^ The inclusion criteria for experts were as follows: be paediatric dentists; have at least 5 years of experience in paediatric dentistry; and be doing the primary tooth pulp treatment on a regular basis. The inclusion criteria for students were as follows: be an undergraduate dental student (of a continuous 6‐year programme) and have passed theoretical and practical paediatric courses.

Following this, we selected eight paediatric dentists in three different cities (Tehran‐Isfahan‐Yazd), four of them with an academic teaching background. For the novices group, we selected eight students from three dental schools in the same cities.

The first researcher conducted one‐to‐one interviews with each of the experts and students via Skype. We sought ethical approval from the Kharazmi University Ethics Committee. Before the start of the interview, each interviewee agreed to participate in the study by signing a consent form. All interviews were audio recorded. Each interview lasted approximately 45 min.

### 2.3 Analysis

We performed a thematic analysis of the data following the six phases outlined by Braun and Clarke^19^: 1. familiarising with the data; 2. generating initial codes; 3. searching for themes; 4. Rrviewing themes; 5. defining and naming themes; and 6. producing the report. All interviews were first transcribed verbatim, reviewed and checked to confirm their accuracy by the first author. Next, two authors extracted initial codes and searched for potential themes, defining and naming them independently and solving any disagreements through discussion. The last author consequently reviewed and analysed all the extracted codes and themes.

## 3 RESULTS

The demographic information showed that the average age of participants in the expert group was 40, and for the novice group, it was 23. On average, the experts had 7 years of work experience and regularly performed pulp therapy for primary tooth with an average number of 50 procedures per year. Moreover, all students had passed the theoretical and practical paediatric courses, five students were in their sixth year and three of them were at the end of their fifth year; on average, they had five cases in primary pulp treatment during clinical practice.

### 3.1 Clinical management

We identified three overarching themes for all data: a child‐related, parent‐related and student‐related theme. Table 1 presents the themes and the factors and errors for each theme that complicate clinical management.

**TABLE 1:** The themes and their complicating factors and typical errors in clinical management

Factors/errors per theme:	Complicating factors:	Typical errors:
1: Child‐related	Non‐cooperative child The child's characteristics	Poor communication
2: Parent‐related	Presence of parents Socio‐cultural and linguistic differences	
3: Student‐related	The challenge between limited time and the need to establish good communication A lack of practical experience or clinical skills, due to overemphasis on theoretical training Insufficient mastery of behavioural management techniques Poor ability to manage emergency situations	Lack of theoretical and practical knowledge (techniques) Unsuccessful application of behavioural management techniques Insistence on treatment Low self‐confidence

#### 3.1.1 Child

All participants (students *and* experts) unanimously considered the level of child collaboration as a factor that effectively mediated the complexity of clinical management. The non‐cooperative child included a continuum of different factors and characteristics such as individual differences, age, special cases (mental and physical disability or disorder), the child's pain or anxiety and the child's previous negative experiences. Sluggish action on the part of the dental student could sometimes also affect the child's cooperation. The second child‐related factor was the child's characteristics, including his or her developmental features, psychological aspects such as shyness, introversion, being unpredictable or aggressive and gag reflex. With respect to typical errors, we identified specifically “poor communication.” Although this error could also be ascribed to the dental student, the child's characteristics and cooperation could complicate this situation, making dental students more prone to errors.

when they [children] always get what they want, they think ‘I am the boss here’, but most of them are scared, and you can't get … help from [the] parents to overcome … this. (EXP‐P07)

#### 3.1.2 Parent

The first factor was the parents' presence: According to most participants, the parents had stress and transferred this to their child. Also, in some cases parents were overprotective, they interfered with the dental student's work, complicating his/her interactions with the patient. Doubts about parental presence or absence arose in cases where the dental student needed further information or assistance from the parent. The second parent‐related factor was socio‐cultural and linguistic differences that could produce differences in attitudes and expectations from treatment, health care, trust and interaction with the dentist.

#### 3.1.3 Student

Participants mentioned four complicating factors, including four common errors, which were related to the dental student, and, more specifically, to his or her theoretical and practical knowledge, training, clinical experience and team working skills. The first factor was the challenge between limited time and the need to establish good communication with the patient. Although there were various tools available to diagnose and determine anxiety and child cooperation, there was no specific protocol on how to initiate interaction with the child. The challenge for students was to find a way to gain the child's trust and interact well with the child before moving on to the next stages of clinical examination. The second factor was a lack of practical experience or clinical skills, due to an overemphasis on theoretical training. Because of the complex nature of the tasks and ethical considerations, students' initial training in clinical management had been restricted to theoretical instruction. Supervisors presented lectures on existing behavioural control techniques, and students had merely acted as observer before their first encounter with real patients.

We read a lot about communication techniques, but we do not have any practical training for this, so when we encounter the child, we do not know how we should use those techniques and which one is proper based on the case. (STU‐P06)

This overemphasis on theory in training also underlay the third factor: Insufficient practice of behavioural management techniques caused students to experience complex situations during their first encounter with the child. The last factor related to students was their poor ability to manage emergency cases, again caused by an overemphasis on basic, theoretical training in emergency care. Moreover, since emergency cases rarely occurred in practice, students easily forgot this information, whilst various other factors, such as stress, lack of experience and insufficient knowledge, could also influence their control of the situation.

With respect to typical errors related to the dental student, participants first mentioned a lack of theoretical and practical knowledge. For instance, some students did not know which developmental characteristics of the child were typical of each age, nor did they know the essential differences between child and adult characteristics. The second error, unsuccessful application of behavioural management techniques, was as discussed above due to a lack of practice. The third error was their insistence on treatment, even in complex cases, such as a non‐cooperative child, where referral to a paediatric dental specialist would be preferred. The last error was low self‐confidence in interacting with the child and parent, in using the behavioural management techniques and in writing a treatment plan.

### 3.2 Clinical reasoning

Table 2 presents the themes and the factors and errors for each theme that complicate clinical reasoning.

**TABLE 2:** The themes and their complicating factors and typical errors in clinical reasoning

Factors/ errors per themes:	Complicating factors	Typical errors
1: Child‐related	1. Diagnosing the painful teeth 2. High salivation 3. Anatomical and physiological differences between children and adults 4. Child's age and tooth development affecting the course of treatment 5. Unwillingness to cooperate 6. Special cases (dental and individual) 7. Lack of oral hygiene	1. Disregarding the child's age and tooth development when writing the treatment plan 2. Misdiagnosing the painful tooth 3. Basing the treatment plan on the wrong information
2: Parent‐related	1. Cultural states: parents' different demands/expectations affected the treatment plan	
3: Student‐related	1. Having difficulties in considering all individual patient aspects when writing the treatment plan 2. Clinical judgement 3. Inadequate radiography (quality and interpretation) 4. Lack of practical experience, due to a focus on theory in training	1. Being unfamiliar with tooth morphology and anatomy 2. Inaccurate clinical judgements 3. Misinterpretation of radiographs 4. Insufficient transfer of learning from theory to practice

#### 3.2.1 Child

The first two factors related to the child's condition and included the correct diagnosis of painful teeth, especially in young children or in cases where several decayed teeth were adjacent. The third and fourth factors concerned anatomical and physiological differences between children and adults and the child's age and tooth development. The last three factors involved the child's willingness to cooperate, special cases and oral hygiene. More specifically, the child's level of collaboration affected the treatment plan; in some cases, for instance, when the child was not willing to undergo radiography, it could lead to a mistake in clinical examination and diagnosis. In cases with special dental and individual needs, moreover, it could be more difficult to write the treatment plan. The last complicating factor related to the child was a lack of oral hygiene. In some cases, where a lack of hygiene or genetic factors had caused high decay or lesions, the decision to treat was difficult because it seemed useless in the absence of care.

As mentioned before, the above‐listed child‐related complicating factors made novices more prone to typical errors in clinical reasoning and in writing the treatment plan.

In some cases, three adjacent teeth need to be treated with a pulpotomy, but determining which of the teeth is the cause of pain and should be treated first is very important. [This can be done] by checking the radiography and [by doing] clinical tests such as precaution and heat and cold tests. (EXP‐P02)

The patient's misinformation may lead to an error, for example, which teeth have pain or is it a referral pain or not? (STU‐P04)

#### 3.2.2 Parent

Then, there were also factors affecting the treatment plan that was related to the different demands/expectations of the parent. More specifically, the social and cultural state of the family could influence the treatment plan of each individual patient, including the parents' occupation and education, the quality of the patient‐family relationship, siblings and parenting style.

#### 3.2.3 Student

The overarching factor was the lack of clinical practice before their first encounter with patients, again pointing to a gap between theory and practice. This was detailed in clinical judgement in general and more in particular how to deal with all (or what) patient aspects when writing an individual treatment plan and how to account for in the treatment plan for indications and contraindications. A specific case was how to deal with inadequate radiography information. In some cases, radiographs that were of low quality did not reflect radiolucency around the root, which could lead to a wrong decision or to misinterpretations of radiographic images.

Finally, the errors related to the dental student included unfamiliarity with tooth morphology and anatomy, inaccurate clinical judgements, misinterpretation of radiographs and, lastly, inadequate clinical experience because of the aforementioned gap between theory and practice.

In the first encounter, most students have problems, like: ‘What questions should I ask the patient?’ and ‘What tests should I do?’ [The presence of a virtual patient and practice before seeing a real patient can increase students' self‐confidence] (STU‐P01)

### 3.3 General findings

In the final part of the interview, we asked participants how students received feedback from their supervisors in each stage of clinical management and clinical reasoning during their training courses. The results showed that the theory‐based courses did include a theoretical evaluation. However, students did not apply the theory in a training setting, and their first practice took place with real patients in the clinic, and here, they received feedback from their supervisors.

Moreover, we asked all participants for their opinion about the feasibility of using simulation to teach students clinical management and clinical reasoning skills. All participants were unanimously in favour of using simulation in the pre‐clinical phase. They stated that this could be very effective in behavioural management training, especially in teaching students how to communicate and interact with patients in paediatric dentistry. Participants acknowledged that the use of simulation in dental education could offer students a novel educational experience introducing students to a variegated range of cases in anatomy and physiology, including emergency and complicated situations, systematic disease, age and tooth development, radiographic images and different interpretations. Also, by offering students training and the opportunity to practise in conditions quite similar to the real environment, simulation would improve self‐confidence, group discussion, writing of the treatment plan for inexperienced students and give the opportunity to receive systematic feedback on “where am I going?”, “how am I going?” and “where to next?”.^20^

## 4 DISCUSSION

Transition of theoretical knowledge and skills to actual clinical practice is a complex part of dental and medical training. Due to teaching methods, that focus mainly on theoretical knowledge most graduate students do not have enough self‐confidence and communication skills when interacting with patients.^21^

As our findings demonstrated, most dental students have many problems in clinical management and clinical reasoning during their first encounters with patients. We identified three main themes underlying their problems, that is related to child, parent or dental student. Whilst some of these difficulties were attributable to the complex nature of these tasks,^14^ others were related to the dental student and their education. Our findings confirmed that students primarily received *theoretical* instruction on child characteristics and that a lack of practical training made them prone to errors. This included the challenge between limited time and the need to establish good communication, the need to consider all individual patient aspects and a lack of practical experience and insufficient mastery of behavioural management techniques to deal with demanding parents or a non‐cooperative child. These factors, in turn, led to errors such as unsuccessful behavioural management and low self‐confidence about the treatment plan. This study was undertaken to investigate the factors that complicate the transfer of knowledge from the classroom to the real clinical setting, including the errors that novices typically make. The factors and errors identified confirm that it is crucial to consider this transfer.^3^, ^10^ Apparently, the separation in teaching of theory and practice and therewith a lack of practice leads to an insufficient integration of knowledge, skills, and attitudes and lack of transfer to the clinical setting. The four‐component instructional design (4C/ID) model addresses this by emphasising the use of authentic learning tasks and providing guidelines for the design of complex learning settings that improve the integration of knowledge, skills and attitudes, the coordination of constituent skills and the transfer of learning to real‐life situations.^6^, ^8^

Recent studiesy^21^ support that transfer can be improved with more innovative methods. Similarly, all participants in our study agreed that the use of simulation could improve dental training and practice. Dental education can benefit from simulation by giving students the opportunity to interact with virtual patients and practise both their communication skills and treatment planning. Our findings can provide valuable data for the design of virtual patients using the complicating factors and common errors identified, as well as participants' suggestions.

## 5 CONCLUSION

This study has provided further insight into the factors that affect general dentistry students' performance on clinical management and clinical reasoning in primary teeth pulpotomy. Despite the complex nature of these tasks and students' inexperience, the results of this study provide evidence that part of these difficulties and errors that are common amongst novices are related to education and to the gap between knowledge and practice. We, therefore, propose the use of simulation as a way to bring theory and practice together and help fill this gap. With the help of simulation, educators can offer learning opportunities with more diversity for students and more safety for patients. However, the way simulation is embedded in education remains crucial and further studies are, therefore, needed to design and evaluate simulation applications in dental education.

## CONFLICT OF INTEREST

The authors have no conflict of interest to declare.

## Data Availability

Data available on request from the authors.
